# Description of physical rehabilitation in intensive care units in Argentina: usual practice and during the COVID-19 pandemic. Online survey

**DOI:** 10.5935/0103-507X.20210026

**Published:** 2021

**Authors:** Matias Nicolás Bertozzi, Sabrina Cagide, Emiliano Navarro, Matias Accoce

**Affiliations:** 1 Sanatorio Anchorena San Martín - Buenos Aires, Argentina.; 2 Hospital Donación “Francisco Santojanni” - Buenos Aires, Argentina.; 3 Hospital Municipal “Dr. Bernardo Houssay” - Vicente Lopez, Argentina.; 4 Centro del Parque Cuidados Respiratorios - Buenos Aires, Argentina.; 5 Hospital General de Agudos “Carlos G. Durand” - Buenos Aires, Argentina.; 6 Hospital de Quemados “Dr. Arturo Umberto Illia” - Buenos Aires, Argentina.; 7 Universidad Abierta Interamericana - Buenos Aires, Argentina.

**Keywords:** Early mobilization, Critical care, Physical therapy modalities, Rehabilitation, Survey and questionnaires, Respiration, artificial, Ambulación precoz, Cuidados críticos, Modalidades de fisioterapia, Rehabilitación, Encuestas y cuestionarios, Respiración artificial

## Abstract

**Objective:**

To describe the usual practice of mobility therapy in the adult intensive care unit for patients with and without COVID-19.

**Methods:**

Online survey in which physical therapists working in an adult intensive care unit in Argentina participated. Sixteen multiple-choice or single-response questions grouped into three sections were asked. The first section addressed personal, professional and work environment data. The second section presented questions regarding usual care, and the third focused on practices under COVID-19 pandemic conditions.

**Results:**

Of 351 physical therapists, 76.1% answer that they were exclusively responsible for patient mobility. The highest motor-based goal varied according to four patient scenarios: Mechanically ventilated patients, patients weaned from mechanical ventilation, patients who had never required mechanical ventilation, and patients with COVID-19 under mechanical ventilation. In the first and last scenarios, the highest goal was to optimize muscle strength, while for the other two, it was to perform activities of daily living. Finally, the greatest limitation in working with patients with COVID-19 was respiratory and/or contact isolation.

**Conclusion:**

Physical therapists in Argentina reported being responsible for the mobility of patients in the intensive care unit. The highest motor-based therapeutic goals for four classic scenarios in the closed area were limited by the need for mechanical ventilation. The greatest limitation when mobilizing patients with COVID-19 was respiratory and contact isolation.

## Abstract (es)

**Objetivo:**

Describir la práctica habitual de asistencia kinésica motora en la unidad de cuidados intensivos de adultos, tanto en pacientes con y sin COVID-19.

**Métodos:**

Estudio observacional transversal de tipo encuesta online. Se incluyeron kinesiólogos que trabajan en unidades de cuidados intensivos de adultos en Argentina. Se realizaron 16 preguntas de respuesta múltiple o simple agrupadas en 3 apartados. El primero caracterizado por datos personales, profesionales o del ámbito laboral. El segundo, destinado a conocer el accionar habitual y un tercero enfocado en las prácticas bajo la pandemia COVID-19.

**Resultados:**

Sobre 351 kinesiólogos, el 76.1% reportó que la movilización de los pacientes estaba a cargo exclusivamente de ellos. El objetivo máximo a alcanzar desde el aspecto motor fue variable según cuatro escenarios: Pacientes en ventilación mecánica, desvinculados de la ventilación mecánica, los que nunca estuvieron asociados a la ventilación mecánica y con COVID-19 en ventilación mecánica. En el primer y último escenario el objetivo máximo fue optimizar valores de fuerza muscular. En los restantes fue realizar actividades de la vida diaria. Por último, la mayor limitante en el abordaje de pacientes con COVID-19 fue el aislamiento respiratorio y/o de contacto.

**Conclusión:**

Los kinesiólogos en Argentina reportaron encargarse de la movilización de los pacientes en la unidad de cuidados intensivos. Los objetivos máximos desde el aspecto motor para cuatro escenarios clásicos en el área cerrada podrían estar determinado por la asociación con la ventilación mecánica. La mayor limitación a la hora de movilizar a pacientes con COVID-19 fue el aislamiento respiratorio y de contacto.

## INTRODUCTION

Patients who are admitted to the intensive care unit (ICU) have an increased likelihood of developing numerous complications.^(1,2)^ These factors lead to more days of mechanical ventilation (MV), longer ICU stays, longer hospital stays and higher mortality rates.^(3-6)^

Since 2018, early mobilization has been included as a pillar of in the management of pain, agitation and *delirium* and the prevention of complications that develop in intensive care.^(7-9)^ The main benefits are recovering muscle strength and physical function and decreasing the number of days of MV, the length of stay in the ICU and hospital, mortality rates and the incidence of *delirium*.^(10-12)^

However, early mobilization faces barriers and limitations related to the staff care, medical supplies, and the respiratory, cardiovascular and/or neurological conditions of the patients.^(10,13)^

In the context of the COVID-19 pandemic and given the need to implement respiratory and contact isolation^(14)^ along with more frequent prone positioning^(15)^ as a strategy for refractory hypoxemia,^(16)^ we believe that both the quality and quantity of mobility intervention could be affected in ways that undermine the previously mentioned benefits.

Currently, early mobilization is guided by the practices of individual institutions, and consensus regarding both terminology and way of implementation is lacking. In this regard, we found no information in the literature regarding usual practices related to early mobilization in Argentina or whether the pandemic has affected its implementation in the ICU.

For this reason, the objective of the present survey was to describe the usual practice of mobility therapy for patients with and without COVID-19 in Argentinian adult ICUs.

## METHODS

A cross-sectional observational study was conducted online from June 1 to June 30, 2020. A bibliographic search was conducted in the MEDLINE database using the terms “*early mobilization*”, “*critical care*”, “*physical therapy*”, “*rehabilitation*” and “*survey*”. Relevant articles in English or Spanish were identified from the obtained results, and those that included information relevant to the rehabilitation process in intensive care were submitted to a full-text review. In turn, bibliographic citations were consulted to expand the possible selection of relevant information. In addition, a semistructured interview of three seniors physical therapists who were specialists in intensive care was conducted to determine variables and relevant questions to include.

Subsequently, the information was summarized, and a first version of the survey was prepared and reviewed by the authors of the study and one physical therapist with more than 10 years of experience in critical care. From this, the second version, consisting of 26 items, was agreed upon. This version was evaluated in a pilot test of 15 subjects who completed the survey and reported on the clarity of the statements along with the time needed to complete it, which varied between 3 and 5 minutes.

After the first draft was completed, the final version consisted of 16 multiple-choice or single-response questions. They were grouped into three sections. The first section pertained to the respondents’ personal, professional or work environment data. The second section asked about the participants’ usual actions in terms of limitations or barriers, maximum treatment goals in different scenarios and measurement tools used to assess changes in patients’ physical condition. The third section addressed mobility therapy in the context of the COVID-19 pandemic (Annex 1). The confidentiality of all information obtained was strictly maintained by the researchers; the participants’ data were protected by the Argentine Personal Data Protection Law No. 25,326 (Habeas data law). All data were collected through a virtual platform (Google forms®) and subsequently anonymized, and access was restricted to authorized personnel for the purposes of the study only to ensure the confidentiality of the information.

Physical therapists working in adult ICUs in Argentina were included. A convenience sample was obtained from a database developed by the study authors. Through nonprobabilistic sampling, physical therapists were invited to participate via email and social networks (WhatsApp^®^, Twitter^®^ and Facebook^®^). No survey was eliminated later because only complete surveys were accepted. The link was shared by three of the researchers, and in cases where no response was obtained via email, it was re-forwarded every week up to a maximum of three times. The present work was approved by the Teaching and Research Committee of the *Sanatorio Anchorena San Martín*.

### Statistical analysis

Categorical variables are presented as absolute numbers and percentages. Continuous variables with a normal distribution are presented as the mean and standard deviation (SD). For the analysis of the data, the statistical program SPSS version 24 (IBM Corp, Armonk, NY, USA) was used.

## RESULTS

From June 1 to 30, 2020, 351 physical therapists in Argentina answered to the survey. Their median age was 34 (IQR 31 - 40) years. A total of 45.3% worked in the Autonomous City of Buenos Aires (CABA), 37.6% worked in the province of Buenos Aires, and the remaining proportion was distributed throughout 19 Argentine provinces (Table 1). The public sphere presented the greatest care burden (57.3%). Among the physical therapists, 76.1% reported that patient mobility is exclusively their responsibility. A total of 23.6% of the centers had mobilization protocols in place in the ICU.

**Table 1:** Workplace where most hours are worked per week

Province/City	n (%)
CABA	159 (45.3)
Buenos Aires	132 (37.6)
Córdoba	8 (2.3)
Mendoza	8 (2.3)
Santa Fe	7 (2)
Santiago del Estero	6 (1.7)
Rio Negro	5 (1.4)
Salta	4 (1.1)
Chubut	3 (0.9)
Jujuy	3 (0.9)
Neuquén	3 (0.9)
Tucumán	3 (0.9)
San Juan	2 (0.6)
Chaco	1 (0.3)
Corrientes	1 (0.3)
Entre Ríos	1 (0.3)
Formosa	1 (0.3)
La Pampa	1 (0.3)
Misiones	1 (0.3)
San Luis	1 (0.3)
Tierra del Fuego	1 (0.3)
Total	351 (100)

CABA - Autonomous City of Buenos Aires.

**Figure 1:**
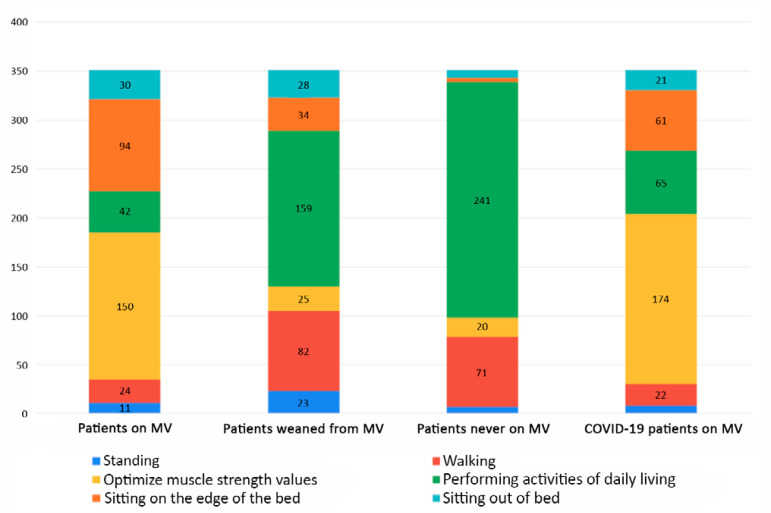
Maximum treatment goals for mobility therapy. MV - mechanical ventilation.

In the second section, concerning the usual actions of physical therapists in Argentina (Table 2), 36.7% reported experiencing no major limitations when mobilizing a patient. The highest reported mobility goal for patients undergoing invasive MV was optimizing muscle strength, followed by sitting on the edge of the bed. The highest mobility goal for patients who have been successfully weaned from invasive MV was performing activities of daily living (ADLs), followed by walking. The highest mobility goal reported for patients who had never required MV was performing ADLs, followed by walking (Figure 1).

**Table 2:** Results for Section 2, "Your actions"

Variables	n (%)
Greatest limitation when mobilizing a patient	
None of the above	132 (37.6)
Pain	89 (25.4)
Physical constraints. catheters. probes and patient-ventilator interfaces	66 (18.8)
Deep sedation	36 (10.2)
Respiratory and/or contact isolation	26 (7.4)
Supplemental oxygen requirement	2 (0.6)
Highest goal for patients under MV	
Optimizing muscle strength	150 (42.7)
Sitting at the edge of the bed	94 (26.8)
Performing activities of daily living	42 (12)
Sitting out of bed	30 (8.6)
Walking	24 (6.8)
Standing	11 (3.1)
Highest goal for patients who have been WEANED from MV	
Performing activities of daily living	159 (45.3)
Walking	82 (23.4)
Sitting at the edge of the bed	34 (9.6)
Sitting out of bed	28 (8)
Optimizing muscle strength	25 (7.1)
Standing	23 (6.6)
Highest goal for patients who NEVER required MV	
Perform activities of daily living	241 (68.7)
Walking	71 (20.2)
Optimizing muscle strength	20 (5.7)
Sitting out of bed	8 (2.3)
Standing	7 (2)
Sitting out of bed	4 (1.1)
Do you use goal-based tools to assess physical condition?	
No	198 (56.4)
Yes	153 (43.6)

MV - mechanical ventilation.

A total of 56.4% of the respondents did not use goal-based validated tools to assess the physical condition of their patients in the ICU. Among those who did use them, the *Medical Research Council* (MRC) and Barthel index were among the most frequently used (Table 3).

**Table 3:** Response to item 11

Variable	n (%)
MRC	129 (71.6)
Other	29 (16.1)
Barthel index	22 (12.3)
Total	180 (100)

MRC - Medical Research Council. For question 11, respondents could select more than one answer.

Regarding the third section (Table 4), which pertained to early mobility therapy in patients with confirmed or suspected COVID-19, 53.7% of the respondents reported decreased intervention compared to usual practice. When asked about their feelings when caring for patients with confirmed or suspected COVID-19, 66.1% reported feeling cautious and selecting timing and interventions as necessary. A total of 49.6% of respondents did not know whether ICU-acquired weakness occurred more frequently in patients with confirmed or suspected COVID-19. The respondents reported that the greatest limitation when mobilizing a patient with confirmed or suspected COVID-19 was respiratory or contact isolation (31.1%), followed by a lack of personal protective equipment (30.8%). Finally, the highest goal reported for patients with confirmed or suspected COVID-19 under MV was optimizing muscle strength (49.6%).

**Table 4:** Results for Section 3, "Impact of the COVID-19 pandemic"

Variables	n (%)
Motor-based intervention for COVID-19/suspected patients	
Decreased	201 (57.3)
Unchanged	132 (37.6)
Intensified	18 (5.1)
Feelings about COVID-19/suspected patients	
Cautious; I am selective about my timing and interventions	232 (66.1)
Calm; I have protective gear	103 (29.3)
Scared; if I could. I would avoid caring for them	16 (4.6)
Greater ICU-acquired weakness in COVID-19/suspected patients?	
I don't know	174 (49.6)
Yes	127 (36.2)
No	50 (14.2)
Limitations when mobilizing a COVID-19/suspected patient	
Respiratory and/or contact isolation	109 (31.1)
Lack of personal protective gear	108 (30.8)
I don't feel there are any limitations	81 (23.1)
Deep sedation	22 (6.1)
Physical constraints. catheters. probes and patient-ventilator interfaces	19 (5.4)
Pain	9 (2.6)
Supplemental oxygen requirement	3 (0.9)
Highest goal for COVID-19/suspected patients on MV	
Optimizing muscle strength	174 (49.6)
Performing activities of daily living	65 (18.5)
Sitting at the edge of the bed	61 (17.3)
Walking	22 (6.3)
Sitting out of bed	21 (6)
Standing	8 (2.3)

MV - mechanical ventilation.

## DISCUSSION

The present survey describes the responses of 351 physical therapists working in adult ICUs in Argentina regarding their usual practice of mobility therapy and how the pandemic has influenced it.

Regarding the proposed treatment goals for the three scenarios, patients receiving MV were assigned the lowest treatment goal, while patients in the other two scenarios were assigned the highest goal (Figure 1). These findings may suggest that for the surveyed physical therapists, MV in itself is a limitation to progressing with different motor-based treatment strategies. These beliefs may be installed in the culture of intensive therapies, which is permeated by the belief that intensive care patients are too sick to tolerate any activity and that functional deterioration is inevitable after a critical illness.^(17)^

For patients receiving MV, the highest motor-based goal was optimizing muscle strength. In South America, Pires-Neto et al. reported that more than half of the activities that were carried out in patients with MV were related to in-bed mobilization.^(18)^ Schweickert et al.^(11)^ described that although preventive mobility therapy did not restore muscle strength, it enabled functional rehabilitation in this group of patients. In line with this, we consider it necessary to review the therapeutic targets proposed for each scenario in future research.

Another factor that was discussed is the probable relationship between the lack of mobilization protocols and the proposed treatment goals. Hanekom and Elliott described the benefits and outcome improvements in centers that use mobilization and analogous sedation protocols compared to those that do not.^(19,20)^ We believe that combining work protocols, the current open ICU concept family empowerment^(21)^ and reviews of the goals for each treatment session could improve the outcomes of critically ill patients.

Regarding barriers or limitations, although the response options provided in the survey reflected the barriers and limitations most frequently reported in the literature,^(22,23)^ our respondents most often selected “none of the above”. A possible explanation for this finding is an error in the wording or interpretation of the question; the respondents may have interpreted this option as indicating the absence of any limitations. It should be clarified that this option was added after the expert review and pilot test.

On the other hand, the participants indicated that the limitations for treating patients with confirmed or suspected COVID-19 were respiratory and/or contact isolation, followed by a lack of personal protective gear, results similar to those reported by Valenzuela et al.^(14)^ It is likely that health personnel in general have become more aware of the importance of personal care, and this awareness forces them to choose the optimal moment for intervention and rely on available material in developing their activities. We believe that in this sense, the pandemic has forced healthcare providers to review how they perform their “usual” and will likely generate changes in work attitudes going forward.

Finally, the MRC and Barthel index scales were the tools most frequently used for assessing the physical condition of patients, consistent with the findings of other local studies.^(24,25)^ Castro-Avila et al.,^(26)^ in their systematic review and meta-analysis, reported that the 6-minute walk test and timed up-and-go test were the most commonly used tools for assessing physical condition upon ICU discharge. These findings suggest that the tools selected by our respondents may not necessarily represent the physical condition of critical patients upon ICU discharge.

The present survey recorded the responses of physical therapists working in Argentina regarding mobility therapy in ICUs. These practices were previously unknown; they have been minimally studied, and the related terminology and results are heterogeneous and nonspecific. In this sense, we believe that the results of our study are valuable for laying the foundations for future research and can deepen and generalize findings in such areas as goal-driven assessment strategies, which in turn will allow possible preventive and/or treatment approaches to be proposed.

As limitations, we can highlight that the multiple-choice response format could have restricted the respondents’ responses. In turn, the recruitment of participants through social networks could have generated selection bias. We believe that with a longer dissemination time, our results could have had greater reach and thus reflected the reality at the national level, rather than mainly focusing on the Autonomous City of Buenos Aires and in the Province of Buenos Aires. Finally, it is necessary to develop prospective studies to compare the functional outcomes of COVID-19-positive patients and patients without COVID-19 at discharge from intensive therapy and in the long term.

## CONCLUSION

The physical therapists surveyed in Argentina reported being responsible for the mobility of patients in the intensive care unit. The highest goal for patients under mechanical ventilation was to optimize muscle strength and make progress towards performing activities of daily living without limitations.

Regarding patients with COVID-19, the greatest limitation for mobilization was respiratory/contact isolation, while the highest goal for patients under mechanical ventilation was optimizing muscle strength.

## Appendix

**Annex 1:** Complete survey

Mobility therapy in critical care during the COVID-19 pandemic
**Section 1 - General data**
1. Age (years)
2. The workplace where you work the highest number of weekly hours is in:
CABA
Buenos Aires
Tierra del Fuego
Santa Cruz
Chubut
Río Negro
Neuquén
La Pampa
San Juan
Córdoba
San Luis
Santa Fe
Entre Ríos
Misiones
Chaco
Formosa
Tucumán
Catamarca
Jujuy
Salta
Mendoza
La Rioja
Corrientes
Santiago del Estero
3. The area with the greatest burden of care is:
Public
Private
4. In your field of work, is patient mobility exclusively handled by physiotherapy?
Yes
No
5. At your center, are there mobilization protocols?
Yes
No
**Section 2 - Your actions**
6. Which of the following do you consider to be the greatest limitation when mobilizing a patient?
Pain
Physical constraints, catheters, probes and patient-ventilator interfaces
Respiratory and/or contact isolation
Deep sedation
Supplemental oxygen requirements
None of the above
7. Which of the following activities do you consider the highest motor-based goal for a patient who is RECEIVING INVASIVE MECHANICAL VENTILATION?
Optimizing muscle strength
Sitting at the edge of the bed
Standing
Sitting out of bed
Walking
Performing activities of daily living
8. Which of the following activities do you consider the highest motor-based goal for a patient who has been WEANED from INVASIVE MECHANICAL VENTILATION?
Optimizing muscle strength
Sitting at the edge of the bed
Standing
Sitting out of bed
Walking
Performing activities of daily living
9. Which of the following activities do you consider the highest motor-based goal for a patient who NEVER required INVASIVE MECHANICAL VENTILATION?
Optimizing muscle strength
Sitting at the edge of the bed
Standing
Sitting out of bed
Walking
Performing activities of daily living
10. In your unit, do you use goal-based tools to assess physical condition?
Yes
No
11. If you answered yes to the previous question, which one(s) do you use?
**Section 3 - Impact of the COVID-19 pandemic**
12. Regarding patients with confirmed or suspected COVID-19, your level of motor-based intervention has
Intensified
Decreased
Not changed
13. How do you feel about caring for this group of patients?
Scared; if I could, I would avoid caring for them
Cautious; I am selective about my timing and interventions
Calm; I have protective gear
14. Do you think that patients with confirmed or suspected COVID-19 develop more ICU-acquired weakness than other patients?
Yes
No
I don't know
15. Which of the following do you consider the greatest limitation when mobilizing a patient with confirmed or suspected COVID-19?
Pain
Physical constraints, catheters, probes and patient-ventilator interfaces
Respiratory and/or contact isolation
Deep sedation
Supplemental oxygen requirements
I don't feel there are any limitations
Lack of personal protective gear
16. What is the highest goal you aim to achieve with a patient with confirmed or suspected COVID-19 who is RECEIVING INVASIVE MECHANICAL VENTILATION?
Optimizing muscle strength
Sitting at the edge of the bed
Standing
Sitting out of bed
Walking
Performing activities of daily living
